# Effects of mitomycin C on the expression of chymase and mast cells in the conjunctival scar of a monkey trabeculectomy model

**Published:** 2009-10-13

**Authors:** Kouhei Okada, Tetsuya Sugiyama, Shinji Takai, Denan Jin, Osamu Ishida, Masanori Fukmoto, Hidehiro Oku, Mizuo Miyazaki, Tsunehiko Ikeda

**Affiliations:** 1 Department of Ophthalmology, Takatsuki Red Cross Hospital, Osaka, Japan.; 2 Department of Ophthalmology, Osaka Medical College, Osaka, Japan.; 3 Department of Pharmacology, Osaka Medical College, Osaka, Japan.

## Abstract

**Purpose:**

To determine the effects of mitomycin C (MMC) on the expression of chymase and mast cells in the conjunctival scar after trabeculectomy.

**Methods:**

Ten eyes of five monkeys were used. Three eyes underwent trabeculectomy with MMC (MMC-treated), four eyes had trabeculectomy without MMC (placebo-treated), and three eyes served as control eyes. Intraocular pressure was measured before and three weeks after surgery. The scores of the degree of conjunctival adhesion were evaluated. Immunohistochemistry was used to analyze the densities of proliferative cell nuclear antigen-positive cells, chymase-positive cells, and mast cells. The ratio of collagen fiber areas to conjunctival and scleral lesions was analyzed by Mallory-Azan staining.

**Results:**

After trabeculectomy, the intraocular pressure reduction of MMC-treated eyes was significantly different from placebo-treated and control eyes (p=0.032, 0.035). The adhesion score of MMC-treated eyes was also significantly lower than that of placebo-treated eyes (p=0.034). Densities of proliferative cell nuclear antigen-positive cells, chymase-positive cells, and areas of collagen fiber in conjunctival and scleral lesions were significantly decreased in MMC-treated eyes, compared with placebo-treated eyes (p=0.034, 0.034, 0.049, respectively). There was a tendency for the density of mast cells to be suppressed in MMC-treated eyes (p=0.157).

**Conclusions:**

Chymase might be involved in one of the mechanisms by which MMC suppresses scar formation after trabeculectomy.

## Introduction

In trabeculectomy, mitomycin C (MMC) maintained good blebs and greatly improved the results of glaucoma surgery [1-3]. However, by making thin blebs with MMC, there is an increased risk of complications such as infectious endophthalmitis [4-6]. As a mechanism of MMC action, it is commonly accepted that inhibiting fibroblast proliferation leads to decreased conjunctival adhesion and maintaining the bleb [7].

When tissues are damaged by surgery in vivo, blood cells are released from blood vessels, and macrophages from monocytes appear in about 12 h, reaching peak numbers around day three. These macrophages activate inflammatory cells, including fibroblasts and lymphocytes. T-cells appear on the fifth day, and after reaching a peak in numbers by the end of two weeks, they are activated into specific T-cells, which release various cytokines to control the activity and proliferation of fibroblasts. Thus, fibroblasts appear and are activated at least 12 h after surgery by macrophages and various cytokines that are released from T-cells [8-10]. In terms of the pharmacokinetics of MMC, the t1/2 in blood doses of 30, 20, and 10 (mg/body) is 50.2, 42.8, and 10.0 minutes, respectively, following intravenous administration [11]. This suggests that the half-life of MMC is short. Because we use MMC at a dosage of less than 1% on the sclera for several minutes in trabeculectomy, its effective lifespan seems to be less than several hours. Therefore, it seems unreasonable that MMC directly suppresses fibroblast adhesion in the conjunctive blebs, since fibroblasts do not appear for at least 12 h after surgery. Other mechanisms must therefore exist whereby MMC suppresses proliferation of fibroblasts.

It is known that mast cells increase in number in the lesion where inflammation occurs after surgery [12]. Mast cells release various kinds of inflammatory mediators such as histamine, serotonin, cytokines, and serine proteases, which play important roles during adhesion formation [13]. Chymase is a chymotrypsin-like serine protease contained in the secretory granules of mast cells. Human mast cells are classified into two groups: tryptase- and chymase-positive mast cells, and tryptase-positive and chymase-negative mast cells. Most mast cells are of the former group in normal conjunctiva, though the latter increases in allergic diseases [14]. Previous reports demonstrated that chymase induces the accumulation of neutrophils, eosinophils, and other inflammatory cells, and promotes fibril formation of type 1 procollagen into collagen fibers [15,16]. We previously reported that chymase stimulated the proliferation of fibroblasts derived from canine Tenon’s capsule, while a chymase inhibitor suppressed the proliferation of the fibroblasts [17]. In this study, we tested the hypothesis that chymase might be involved in the mechanism of bleb maintenance by MMC, using monkey eyes.

## Methods

### Animals

Five monkeys (Macaca fascicularis) weighing 5.1 kg–10.0 kg were purchased from Keari Co. (Osaka, Japan). Monkeys were housed in an air-conditioned room at approximately 23 °C and 60% humidity with a 12h:12h light-dark cycle. Experimental procedures for animals were conducted in accordance with the ARVO Statement for Use of Animals in Ophthalmic and Vision Research.

### Surgical technique

Monkeys were anesthetized with ketamine hydrochloride (10 mg/kg subcutaneously) (Daiichisankyo Prophama Co., Tokyo, Japan) and maintained with pentobarbital (35 mg/kg intravenously; Dainippon Sumitomo Pharma Co., Osaka, Japan). In the conjunctival sclera flap model, a 10 mm fornix-based conjunctival flap and a 3 mm triangle of single scleral flap were created. A sponge (M.Q.A. sponge; Inami Co., Tokyo, Japan) treated with MMC, and diluted to 0.04% in distilled water, was placed between the conjunctiva and sclera for 5 min. The scleral incision was closed with 10.0 nylon sutures and the conjunctival incision was closed with 9.0 silk sutures.

Seven eyes from four monkeys underwent trabeculectomy: MMC was applied to three eyes of two monkeys (MMC-treated eyes), and a placebo was applied to four eyes of three monkeys (placebo-treated eyes). The remaining three eyes of two monkeys, which did not undergo trabeculectomy, served as control eyes. The intraocular pressure (IOP) of each eye was measured before, and three weeks after, surgery. The IOP was measured with a calibrated pneumatonometer (Model 30 Classic, Medtronic Solan, Jacksonville, FL, USA) under general anesthesia in a face-up position. Three weeks after surgery, monkeys were sacrificed by injecting a lethal dose of potassium chloride, and the degree of adhesion was assessed. The flap (5×10 mm^2^) was harvested without detaching the conjunctival and scleral lesions for histological analysis.

### Adhesion scores

The adhesion degree was determined by comparing the percent of the area of adhesion to the whole flap area (5×10 mm^2^) as an index. Scores were determined as follows: Score 1, the flap could be detached by lightly lifting the flap, or the area of adhesion was not more than 20%; Score 2, the area of adhesion was between 20 and 40%; Score 3, the area of adhesion was between 40% and 60%; Score 4, the area of adhesion was between 60% and 100%

### Histology and immunohistochemistry

Conjunctival and scleral tissue specimens were fixed with 10% buffered formalin and embedded in paraffin. Sections with a thickness of 5 µm were cut, mounted on silanized slides (Dako Japan, Kyoto, Japan), and deparaffinized with xylenes and a series of graded ethanol solutions. Each section was stained with azan and toluidine blue to identify collagen fibers and mast cells, respectively.

Immunohistochemical analysis was performed on at least two paraffin blocks of resected conjunctival and scleral tissue per monkey. To retrieve the antigen, sections were pretreated with 10 mM citrate buffer, pH 6.0, and autoclaved for 15 min at 120 °C, before immunohistochemical staining with proliferative cell nuclear antigen (PCNA) and chymase antibodies. Sections were soaked in absolute methanol containing 0.3% hydrogen peroxide for 30 min at room temperature to remove endogenous peroxidase activity. To suppress non-specific binding, sections were incubated with 1.5% non-immune goat serum for 20 min. Sections were then incubated with mouse monoclonal antibody 2D11G10D (Katakura Industries Co., Saitama, Japan) against human chymase for 60 min at room temperature. This antibody can detect human chymase in formalin-fixed, paraffin-embedded tissue sections [18]. After washing in phosphate-buffered saline (PBS), the slides were subsequently incubated with biotin-conjugated goat anti-mouse immunoglobulin (Ig) G antibody for 30 min. After washing with PBS, the sections were then incubated with avidin-biotin-peroxidase complex (Dako Japan) for 30 min and washed once more with PBS. Finally, sections were incubated with 0.03% hydrogen peroxide and 0.05% 3,3-diaminobenzidine. The slides were then washed in running tap water, counterstained with hematoxylin, and mounted in Canadian balsam. Non-immunized mouse IgG was used as a negative control. No significant immunohistochemical reactions occurred in the control sections.

### Measurement of fibrotic areas

One slice obtained from each specimen was Mallory-Azan stained, and the blue area was defined as the fibrotic area. In each specimen, the ratio of the fibrotic area to the total area at 40× magnification was measured using a computerized morphometric system (VHX Digital Microscope, Keyence Co., Osaka, Japan).

### Measurement of PCNA-positive cells, chymase-positive cells, and mast cells

We counted PCNA-positive cells, chymase-positive cells, and mast cells at the sites where they accumulated in the conjunctival and scleral lesions, using a light microscope (number per 100× field). The average number of PCNA-positive cells and chymase-positive cells and mast cells in five selected fields was calculated.

### Statistical analysis

IOPs were evaluated by Student’s t-test for unpaired data. Adhesion scores, densities of PCNA-positive cells, chymase-positive cells, mast cells, and collagen areas were evaluated by the Mann-Whitney U test. Differences were considered statistically significant at p<0.05.

## Results

### IOPs before and after surgery

**Figure 1:**
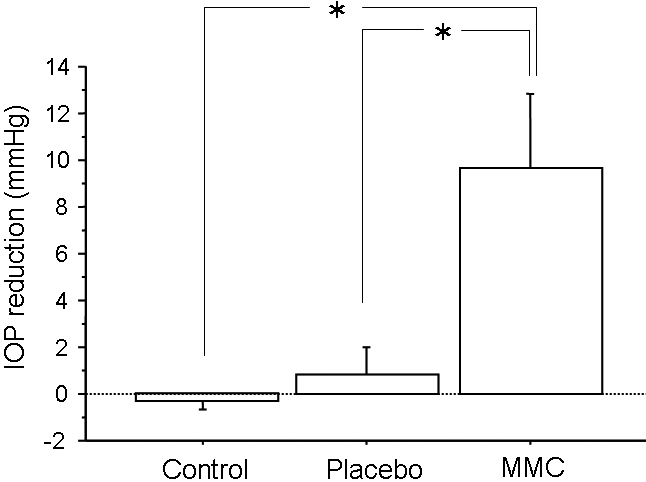
Reduction in intraocular pressure three weeks after trabeculectomy in control, placebo-, and mitomycin C (MMC)-treated eyes. Values are represented as means±SE. The asterisk indicates p<0.05 compared with placebo-treated or control eyes.

Before surgery, the IOP (mean±standard error of the mean (SE), mmHg) was 19.0±2.0 in the control eyes, 18.5±2.5 in the placebo-treated eyes, and 19.0±1.0 in the MMC-treated eyes. There was no significant difference among these groups. IOP reduction after surgery in MMC-treated eyes was significantly different from placebo-treated or control eyes (p=0.0317, 0.0353, Figure 1).

### The adhesion score

**Figure 2:**
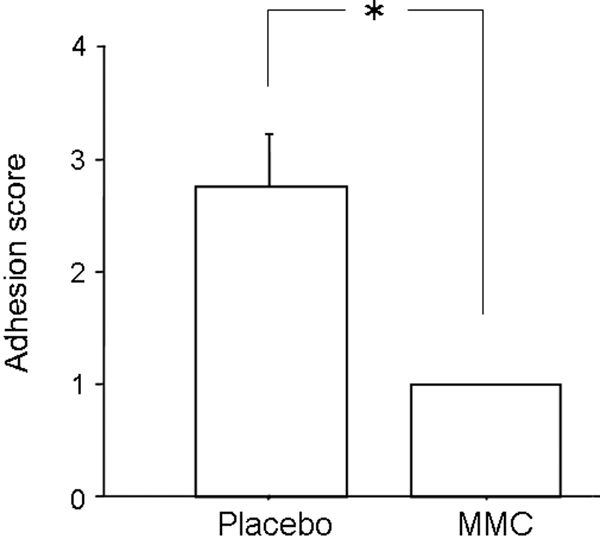
Scores of adhesion formation assessed three weeks after trabeculectomy in placebo- and mitomycin C (MMC)-treated eyes. Values are represented as means±SE. The asterisk indicates p<0.05 compared with placebo-treated eyes.

The adhesion score in MMC-treated eyes was significantly decreased compared with placebo-treated eyes (p=0.0339, Figure 2).

### Histologic study

**Figure 3:**
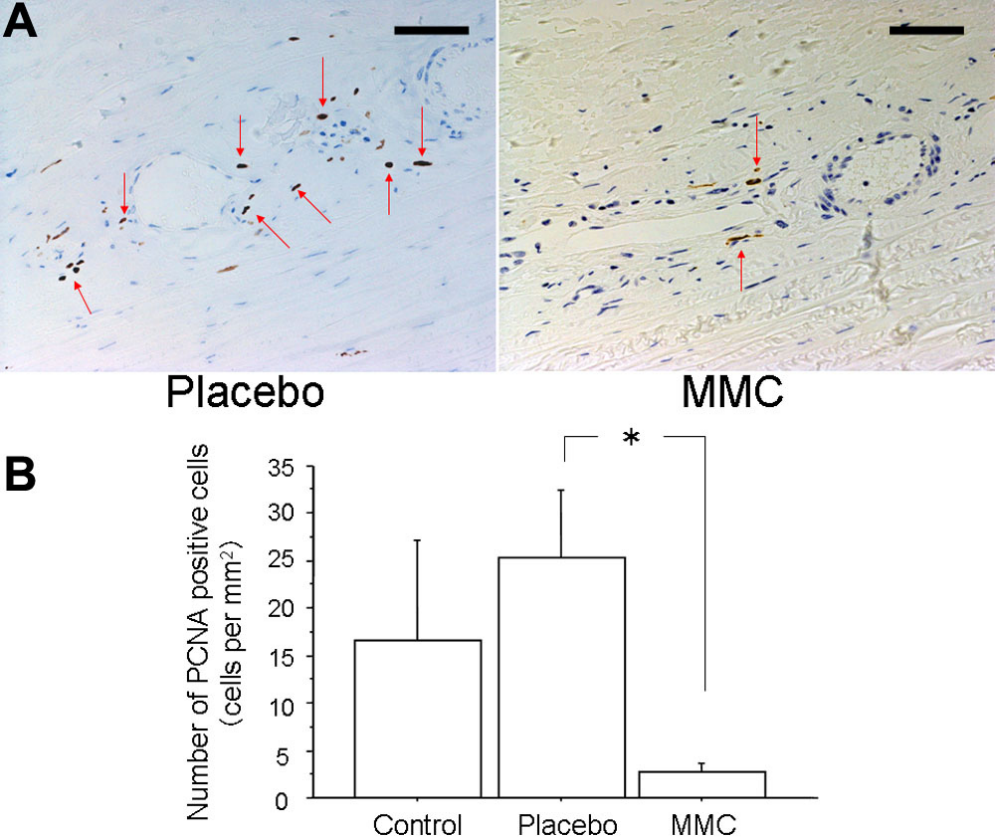
Immunohistochemistry for proliferative cell nuclear antigen (PCNA). **A**: Typical photographs of placebo- (left) and mitomycin C (MMC)- (right) treated eyes stained with PCNA three weeks after trabeculectomy. Bars represent 100 μm. Arrows demonstrate positive cells. **B**: The numbers of proliferative cell nuclear antigen-positive cells per mm^2^ in control, placebo-, and MMC-treated eyes are shown. Values are represented as means±SE. The asterisk indicates p<0.05 compared with placebo-treated eyes.

**Figure 4:**
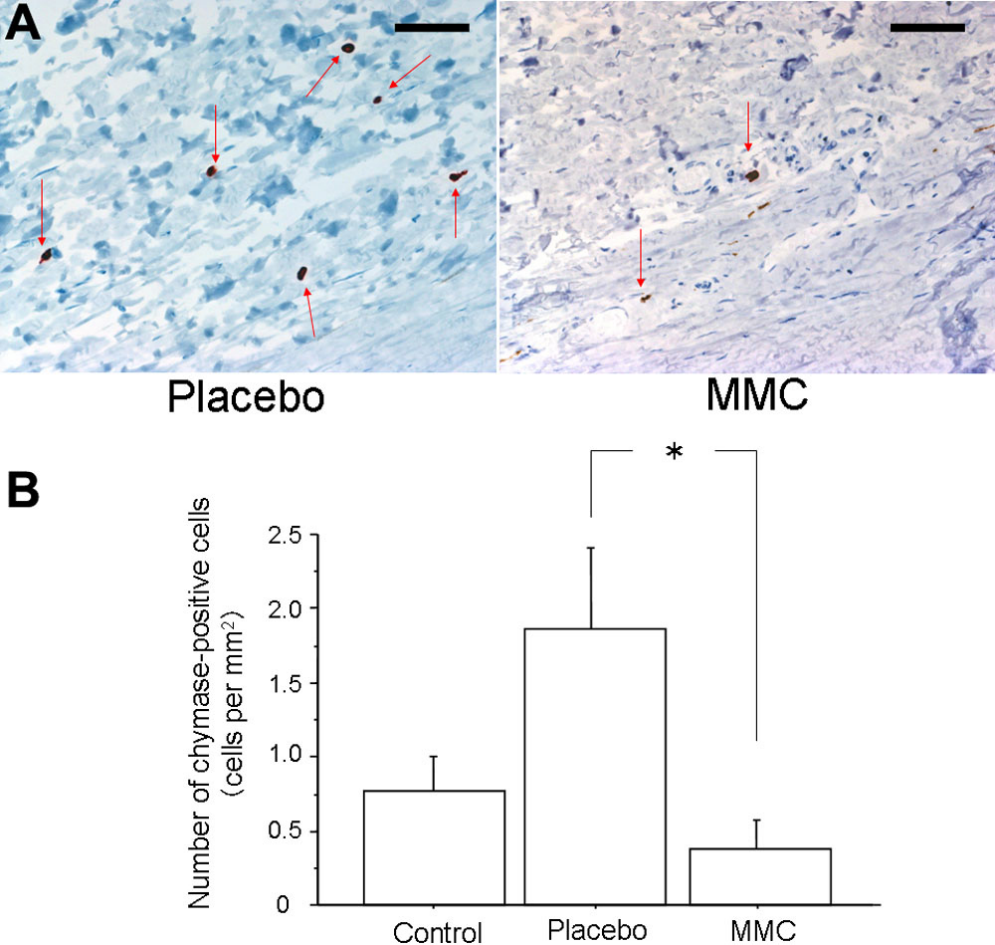
Immunohistochemistry for chymase. **A**: Typical photographs of placebo- (left) and mitomycin C (MMC)- (right) treated eyes stained with chymase antibodies three weeks after trabeculectomy. Bars represent 100 μm. Arrows demonstrate positive cells. **B**: The numbers of chymase-positive cells per mm^2^ in control, placebo- and MMC-treated eyes are shown. Values are represented as means±SE. The asterisk indicates p<0.05 compared with placebo-treated eyes.

PCNA -positive cells in MMC-treated eyes were significantly decreased compared to placebo-treated eyes (p=0.0339, Figure 3). The number of chymase-positive cells in placebo-treated eyes had a tendency to be greater than that in control eyes (p=0.1649), while that in MMC-treated eyes was significantly decreased compared with placebo-treated eyes (p=0.0339, Figure 4). There was no significant difference in the number of mast cells among the three groups. However, the number of mast cells had a tendency to be greater in placebo-treated eyes than in control eyes (p=0.3545), while mast cells had a tendency to be suppressed in MMC-treated eyes compared with placebo-treated eyes (p=0.1573, Figure 5). Collagen fiber areas in MMC-treated eyes were significantly decreased compared with placebo-treated eyes (p=0.0495, Figure 6).

**Figure 5:**
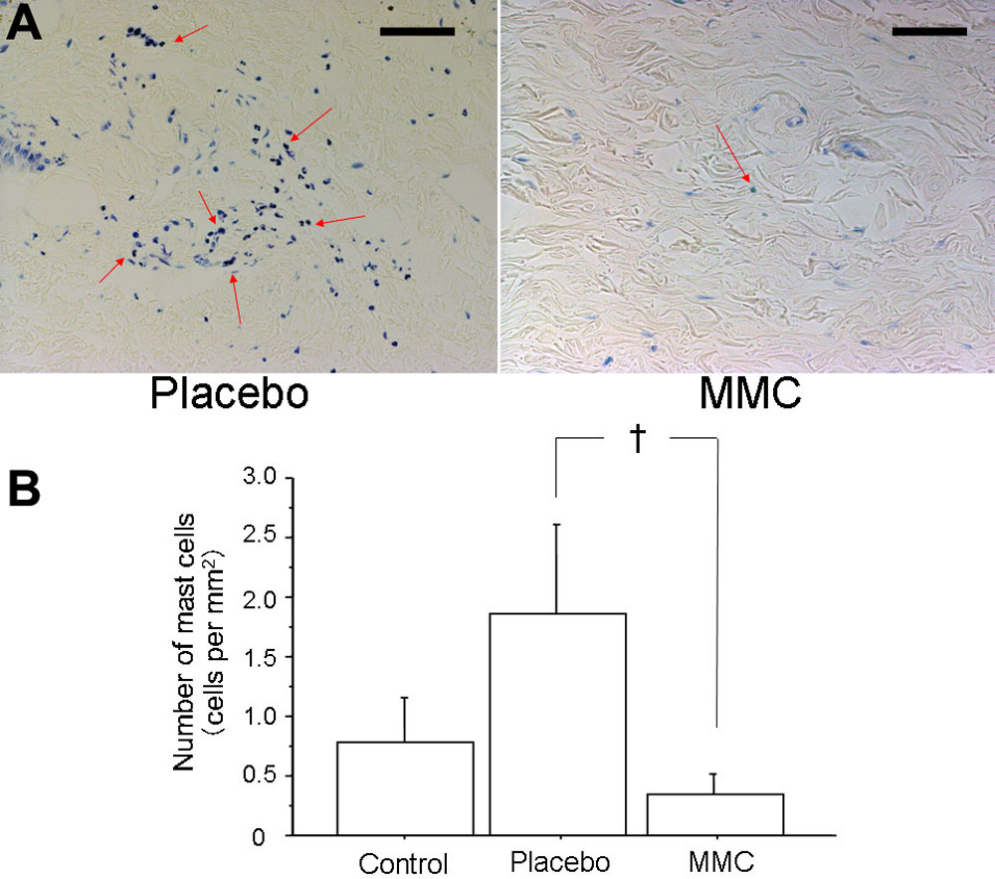
Detection of mast cells. **A**: Typical photographs of placebo- (left) and mitomycin C(MMC)- (right) treated eyes stained with toluidine blue three weeks after trabeculectomy. Bars represent 100 μm. Arrows demonstrate positive cells. **B:** The numbers of mast cells per mm^2^ in control, placebo- and MMC-treated eyes are shown. Values are represented as means±SE. The symbol (†) indicates p<0.2 compared with placebo-treated eyes.

**Figure 6:**
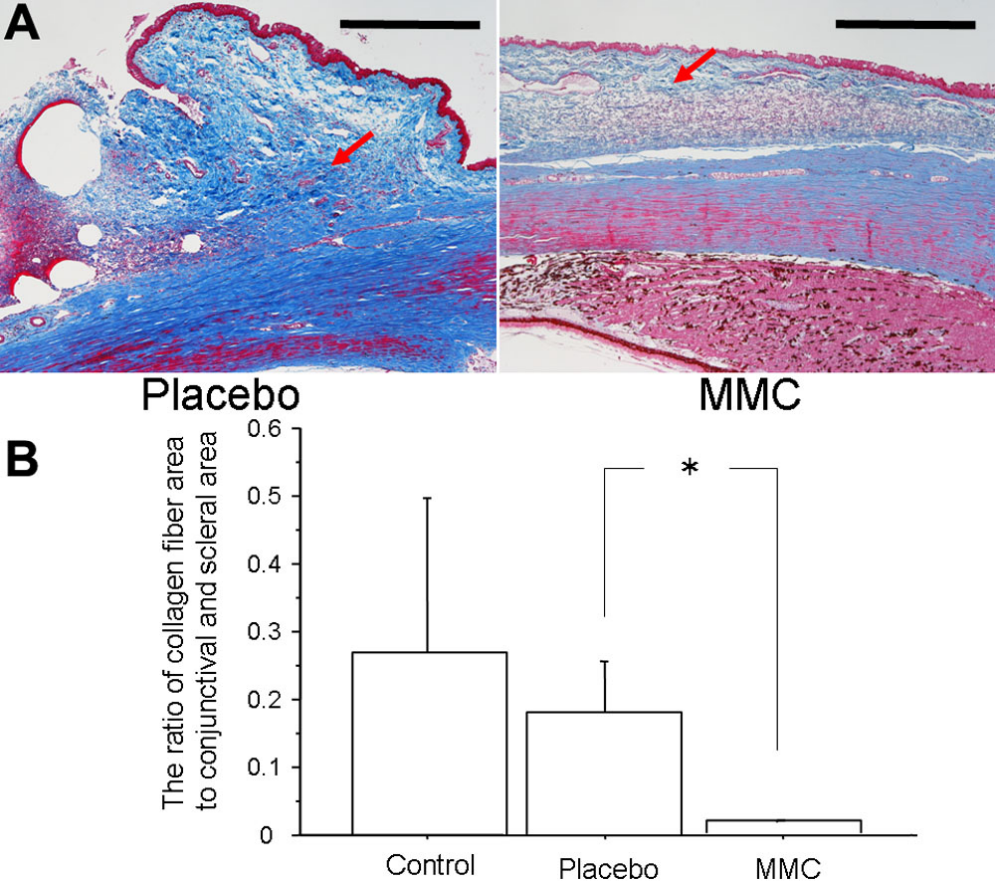
Detection of collagen fibers. **A**: Typical photographs of placebo- (left) and mitomycin C(MMC)- (right) treated eyes stained with azan three weeks after trabeculectomy. Bars represent 500 μm. Arrows demonstrate collagen fibers. **B**: The ratios of collagen fiber areas to conjunctival and scleral lesions in control, placebo and MMC-treated eyes are shown. Values are represented as means±SE. The asterisk indicates p<0.05 compared with placebo-treated eyes.

## Discussion

The results of the current study show that chymase-positive cells in MMC-treated monkey eyes after trabeculectomy were significantly decreased, compared with placebo-treated eyes. Mast cells also tended to be suppressed in MMC-treated eyes. These results suggest that chymase may be involved in at least one of the mechanisms by which MMC exerts its effects.

In this study, the numbers of chymase-positive cells and mast cells had a tendency to be increased in placebo-treated eyes, compared with control eyes, though the differences were not significant. These findings suggest that chymase and mast cells are induced by glaucoma surgery. These results agree with our previous report using canine eyes, even though the animal species were different [17]. In MMC-treated eyes, the adhesion score was suppressed; and PCNA-positive cells, which show changes from non-proliferating cells to proliferating cells, were significantly decreased, in comparison with placebo-treated eyes. These findings suggest that MMC works against proliferation and adhesion after glaucoma filtration surgery, as some other studies have reported [1-3,7,19,20]. Compared to placebo-treated eyes, collagen fiber areas of MMC-treated eyes were significantly decreased, whereas other reports have suggested that collagen increases after glaucoma surgery [21,22]. In comparison to placebo-treated eyes, chymase-positive cells of MMC-treated eyes were significantly decreased. Nevertheless, there are no reports that MMC is directly related to chymase. Mast cells in MMC-treated eyes had a tendency to be suppressed, compared with placebo-treated eyes, although there are no reports that show a relationship between MMC and mast cells. Since chymase exists in the secretory granules that mast cells release [15], it is not inconsistent that mast cells are also decreased as chymase decreases. Nevertheless, the results showing that chymase-positive cells were significantly inhibited, although the suppression of mast cells was not significant, suggest that MMC might have a relatively selective effect on chymase.

It was previously reported that chymase was involved in the adhesion formation of tissues, in terms of fibroblast proliferation [23]. In cultured human fibroblasts, the concentration of transforming growth factor-β (TGF-β) in the medium was significantly increased after the injection of human chymase. Therefore, human chymase increased the cell proliferation of human fibroblasts through activation of the latent TGF-β. The same report also suggested that chymase induced the expression of collagen I and collagen III genes in cardiac tissues, which leads to fibrosis. In a hamster experimental model of peritoneal adhesion, the scores for adhesion formation, chymase activity, and TGF-β levels after surgery were significantly increased in the placebo-treated group, while they were significantly suppressed by treatment with chymase inhibitor [24]. Furthermore, in a hamster model of glaucoma filtration surgery, chymase activity tended to be higher than in control eyes, and mast cells were increased in the subconjunctival tissue and around the tunnel opening [25].

We also reported that chymase promoted cell proliferation of fibroblasts using canine Tenon’s capsule, while chymase inhibitor suppressed proliferation [17]. Other in vitro studies put forward the concept of targeting TGF-β signaling, which is induced by chymase, to prevent scar formation after filtering glaucoma surgery. Activation of the PI3K-Akt pathway by TGF-β2 may be essential for the expression and activity of tissue transglutaminase in subconjunctival fibroblasts. This pathway may play an important role in the pathogenesis of wound healing and fibrogenic reactions in subconjunctival tissue [26]. Another study reported the association of filtering bleb scarring with an abundant expression of TGF-β receptors in activated fibroblasts and the deposition of the fibrogenic ED-A fibronectin splice-variant [27].

Chymase is immediately released from mast cell granules upon tissue damage, such as from surgery, and binds to the extracellular matrix, where it continues to function for several weeks [28,29]. However, MMC probably does not affect the proliferation of fibroblasts appearing around 12 h after tissue damage, because the half-life of MMC is less than 1 h. It is more likely that MMC initially suppresses the proliferation of mast cells, including chymase-positive cells. As a result of this, fibroblast proliferation is then restrained. In fact, topical instillation of an anti-mast-cell agent, tranilast, was useful for filtering bleb formation and IOP reduction [30]. Therefore, suppression of mast cells might be related to formation of the filtering bleb. In addition, chymase inhibition might play a role in maintaining filtering blebs for an extended period of time. It has been reported that a chymase inhibitor prevents adhesion for up to three months in an abdomen adhesion model [31], so bleb formation may be maintained for a long time if MMC inhibits chymase function after tissue injury.

In conclusion, the current study suggests that chymase might be involved in one of the mechanisms by which MMC suppresses scar formation after trabeculectomy. Therefore, inhibition of chymase might be useful for reducing conjunctival adhesion and maintaining bleb formation. Further investigation is needed to verify that chymase inhibitors are appropriate for glaucoma surgeries.
